# Exoproteome Perspective on the Bile Stress Response of *Lactobacillus johnsonii*

**DOI:** 10.3390/proteomes9010010

**Published:** 2021-02-10

**Authors:** Bernadette B. Bagon, Valerie Diane V. Valeriano, Ju Kyoung Oh, Edward Alain B. Pajarillo, Ji Yoon Lee, Dae-Kyung Kang

**Affiliations:** 1 Department of Animal Resources Science, Dankook University, Cheonan 31116, Korea; dette.bagon@gmail.com (B.B.B.); vdvaleriano@gmail.com (V.D.V.V.); supajuko@gmail.com (J.K.O.); eabpajarillo@gmail.com (E.A.B.P.); 2 Center for Food and Bioconvergence, Seoul National University, Seoul 08826, Korea; jiyoon@snu.ac.kr

**Keywords:** extracellular proteome, exoproteome, *Lactobacillus johnsonii*, lactobacilli, bile stress, bile response

## Abstract

Probiotics must not only exert a health-promoting effect but also be capable of adapting to the harsh environment of the gastrointestinal (GI) tract. Probiotics in the GI tract must survive the cell wall-disrupting effect of bile acids. We investigated the exoproteome of *Lactobacillus johnsonii* PF01 and C1-10 under bile stress. A comparative analysis revealed the similarities between the two *L*. *johnsonii* exoproteomes, as well as their different responses to bile. The large number of metabolic proteins in *L*. *johnsonii* revealed its metabolic adaptation to meet protein synthesis requirements under bile stress. In addition, cell wall modifications occurred in response to bile. Furthermore, some extracellular proteins of *L*. *johnsonii* may have moonlighting function in the presence of bile. Enolase, L-lactate dehydrogenase, glyceraldehyde-3-phosphate dehydrogenase, triosephosphate isomerase, 50s ribosomal protein L7/L12, and cellobiose-specific phosphotransferase system (PTS) sugar transporter were significantly upregulated under bile stress, suggesting a leading role in the collective bile stress response of *L*. *johnsonii* from its exoproteome perspective.

## 1. Introduction

*Lactobacillus* species are gram-positive, rod-shaped, non-spore–forming, anaerobic to facultative anaerobic lactic acid bacteria that are generally regarded as safe (GRAS). Their GRAS status is based on their beneficial effect on health. Their ability to produce lactic acid and prevent microbial spoilage led to their use in food fermentation and preservation, as well as for treating enteric diseases (e.g., diarrhea, inflammatory bowel disease, and colorectal cancer). For these reasons, numerous *Lactobacillus* strains have been manufactured as probiotics [1,2,3].

According to Lebeer [2], the performance of lactobacilli as probiotics depends on their ability to adapt to and colonize the gastrointestinal (GI) tract. This is facilitated by resistance to stress, an active metabolism adapted to the host environment, and adherence to the intestinal mucosae and mucus. Thus, one of the prerequisites for probiotics is the ability to tolerate bile, and thus, to colonize the GI tract [4].

Bile acids are synthesized in the liver and transported to the intestine to emulsify or solubilize indigestible lipids. They also affect cell membrane phospholipids and proteins, disrupting the cellular homeostasis of microorganisms in the GI tract. Bile affects the adhesion of bacterial species. Because bile disrupts the cell membrane, it can decrease survivability in the GI tract or expose proteins that promote adhesion and aggregation [5,6,7,8]. These proteins may promote colonization by lactobacilli by participating in the host–microbe interaction.

Lactobacilli in the GI tract have several mechanisms of bile tolerance ranging from single proteins to entire pathways [2]. Such intracellular, surface, and extracellular proteins mediate adaptation to the stressful bile-enriched intestinal environment. Extracellular bacterial proteins directly interact with intestinal epithelial cells [9]. Interestingly, some extracellular proteins are cytoplasmic proteins lacking signal peptides or surface retention domains. Although the mechanisms of the extracellular interactions are unclear, the mediating proteins are believed to be moonlighting proteins with multiple functions in the host–bacterium interaction [10].

Two *Lactobacillus johnsonii* strains, originally isolated from pig intestine (PF01) and chicken intestine (C1-10), demonstrate characteristics that contribute to their potential as probiotics. The PF01 strain is highly tolerant of bile stress [11], and adheres to porcine mucin and intestinal epithelial cells [12], allowing it to persist in the host GI tract. Its genome sequencing revealed three bile salt hydrolases, which hydrolyze all types of bile salts [13]. The C1-10 strain also showed high tolerance to bile (data not shown). During our investigation of PF01 exoproteome in the absence of stress factors, C1-10 was observed to have quite similar exoproteome with PF01, amidst their different initial niches [14]. As potential probiotics, we plan to further investigate the two strains based on their bile stress responses. In this study, we determined the extracellular proteome of two *L*. *johnsonii* strains under bile stress via label-free quantification of protein expression using UHPLC/HESI-tandem mass spectrometry.

## 2. Materials and Methods

### 2.1. Bacterial Strains and Growth Conditions

*Lactobacillus johnsonii* strains from porcine feces (PF01) and chicken intestine (C1-10) were compared in this study. *L*. *johnsonii* PF01 and C1-10 were cultured in de Man-Rogosa Sharpe broth (Difco, Le Pont de Claix, France) at 37 °C under static anaerobic conditions. Seed cultures for each strain were prepared in 100 mL of broth and incubated for 24 h.

### 2.2. Bile Treatment and Extracellular Protein Collection

The procedures from [15] were modified for this study. Cultures were inoculated in 500 mL of broth (1%) and incubated at 37 °C until mid-logarithmic phase (optical density at 600 nm, 0.7 to 0.8). Ox gall or bile bovine (B3883, Sigma-Aldrich, Yongin, Korea) solutions (0.1%, and 0.3%) (*v*/*v*) were added, and the cultures were incubated at 37 °C for 1.5 h. A control lacking ox gall solution (0.0%) was prepared for each strain. Three independent replicates were prepared.

Supernatants containing extracellular proteins were harvested by centrifugation (8000 rpm, 10 min, 4 °C) and passed through a cellulose acetate membrane (0.22 μm pore size) in a half-atmospheric-pressure vacuum. The filtrates were saturated to 80% saturation by ammonium sulfate precipitation overnight (12 h) at 4 °C. The proteins were precipitated by centrifugation (3000× *g*, 20 min, 4 °C) and dissolved in 20 mM sodium citrate buffer (pH 5.0). Dialysis was performed using a regenerated cellulose dialysis membrane (Standard Grade, Spectra/Por) with a 1 kD cut-off and 25 mM phosphate buffer (pH 7.0, 4 °C). The buffer was replaced twice at 2 h intervals, and the equipment was equilibrated overnight (12 h). Protein concentration was determined by Bradford protein assay. Proteins were resolved by sodium dodecyl sulfate-polyacrylamide gel electrophoresis using a 12% resolving gel. In-solution digestion was then performed as described previously [16].

### 2.3. Ultra High-Performance Liquid Chromatography/Heated Electrospray Ionization-Tandem Mass Spectrometry Analysis

Mass spectrometry was performed as described previously [14]. Tryptic digests were separated by reversed-phase chromatography and an ultra-high-performance liquid chromatography (UHPLC; Dionex Ulti-Mate^®^ 3000 (Thermo Fisher Scientific, Seoul, Korea)) instrument with an Acclaim PepMap 100 trap column (100 μm × 2 cm, nanoViper C18, 5 μm, 100 Å); an Acclaim PepMap 100 capillary column (75 μm × 15 cm, nanoViper C18, 3 μm, 100 Å) was used to separate peptides. The UHPLC was coupled to a heated electrospray ionization source (HESI-II) and the quadrupole-based Q Exactive™ Orbitrap High-Resolution Mass Spectrometer (Thermo Fisher Scientific) to generate mass spectra. Fractions were reconstituted in solvent A (water/acetonitrile, 98:2 *v*/*v*; 0.1% formic acid), washed with 98% solvent A for 6 min at a flow rate of 6 μL/min, and then continuously resolved at a flow rate of 400 nL/min. The LC analytical gradient was run from 2% to 35% solvent B (0.1% formic acid in acetonitrile) over a period of 90 min, after which the concentration was increased over 10 min from 35% to 95%. The samples were run on 90% solvent B for 5 min. Finally, solvent B was decreased to 5% and run for 15 min. The peptides were electrosprayed through a coated silica tip (PicoTip emitter, New Objective) at an ion spray voltage of 2000 eV. MS spectra were acquired at a resolution of 70,000 (200 *m/z*) in a mass range of 350–1800 *m/z*. The maximum injection time was set to 100 ms for ion accumulation. Eluted samples were used for succeeding MS/MS events. Ion activation/dissociation was performed using the high-mass-accuracy Orbitrap with Higher Energy C-trap Dissociation at a collision energy of 27 in a 100–1650 *m/z* mass range. Measurement of intensities was performed in data-dependent mode to identify the most abundant peaks (Top10 method).

### 2.4. MS Data Processing and Bioinformatics Analysis

The MaxQuant search engine was used to cross-reference the raw quantified proteins to the available proteome database in UniProt [17]. Proteins of *L*. *johnsonii* PF01 and C1-10 were initially annotated using the protein database for *L*. *johnsonii* NCC 533 (UniProt UP000000581), and then matched to the predicted proteins for the complete genome sequence of PF01 (GenBank GCA_000219475.3) using the BLAST function of CL Genomics Software (ChunLab). Subsequently, the functional annotations of the matched proteins based on Cluster of Orthologous Groups (COG) were derived from the complete genome and used to assign the proteins to their respective functions. In addition, LocateP v2.0 [18] and SignalP4.0 [19] were used with the default settings and cut-off values to determine the localizations and secretion pathways of the protein.

### 2.5. Statistical Analysis of the Effects of Bile

The expression intensities based on label-free quantification (LFQ) of the proteins were log_2_ transformed and subjected to two-way analysis of variance (ANOVA) (GraphPad Prism 8.4.2). To determine which protein expression were significantly different, Tukey’s multiple comparisons test was applied, with significance criteria set at *p* < 0.05 [20]. A heat map of significantly expressed proteins was constructed using the log_2_-transformed values. Proteins showing zero intensities in one or more treatments were considered not detected and/or not expressed and did not need to be log_2_-transformed for comparison.

## 3. Results

### 3.1. Characteristics of Lactobacillus Johnsonii Bile Response Exoproteome

**Figure 1:**
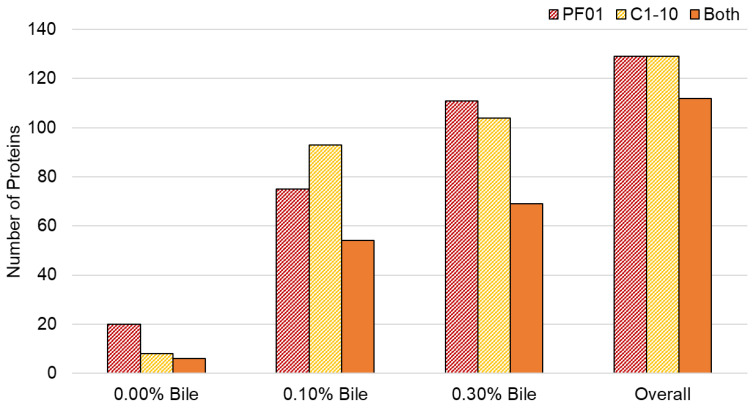
Total proteins detected in *Lactobacillus johnsonii* PF01 and C1-10 exoproteome during bile treatment. Proteins found in both PF01 and C1-10 are listed in Supplementary Table S1.

The bile response exoproteome of *L*. *johnsonii* PF01 and C1-10 was evaluated in this study. Figure 1 shows that both PF01 and C1-10 have 112 extracellular proteins. Bile (0.10% and 0.30%) increased the number of proteins, indicating that secretion of these proteins is a response to bile stress. In addition, common proteins between PF01 and C1-10 were identified and the effect of bile on their levels was evaluated (Supplementary Table S1).

**Figure 2:**
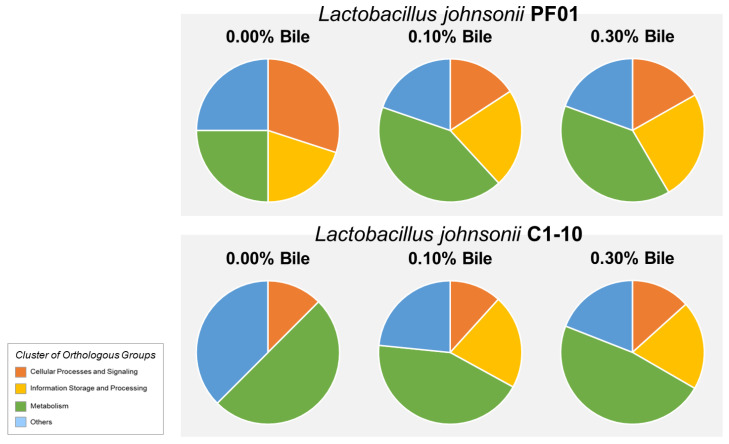
Exoproteome functional composition based on Cluster of Orthologous Groups of *Lactobacillus johnsonii* PF01 and C1-10 proteins during increasing bile treatment.

The identified extracellular proteins were annotated based on COG and classified under four main functional categories: (1) cellular processes and signaling, (2) information storage and processing, (3) metabolism, and (4) others. Most of the proteins were predicted to have metabolic functions (Figure 2). Bile stress increased the number of proteins in the metabolism category in both PF01 and C1-10. Proteins for information storage and processing (translation, transcription, and ribosomal synthesis) were increased by bile, most evidently in C1-10.

The extracellular proteins were classified based on localization (Table 1). Interestingly, based on subcellular localization and cellular destination, most PF01 and C1-10 proteins were predicted to be ‘cytoplasmic’ or ‘intracellular.’ However, not all ‘secreted’ proteins were accounted for by the two categories. It is possible that some ‘cytoplasmic’ proteins harbor secretion domains. Therefore, *L*. *johnsonii* PF01 and C1-10 respond to bile stress by secreting numerous cytoplasmic proteins with metabolic and translational functions.

### 3.2. Species- and Strain-Specific Bile-Induced and Upregulated Proteins

In PF01 and C1-10, enolase, phosphoglycerate kinase (pgk), and pyridoxamine 5′-phosphate oxidase (COG3576; *pyr*) were upregulated by bile but N-acetylmuramoyl-L-alanine amidase (FlgJ) and surface protein/aggregation promoting factor (*SPapf*) were downregulated (Table 2). Forty proteins were induced by bile (0.10% or 0.30%) in both PF01 and C1-10.

**Figure 3:**
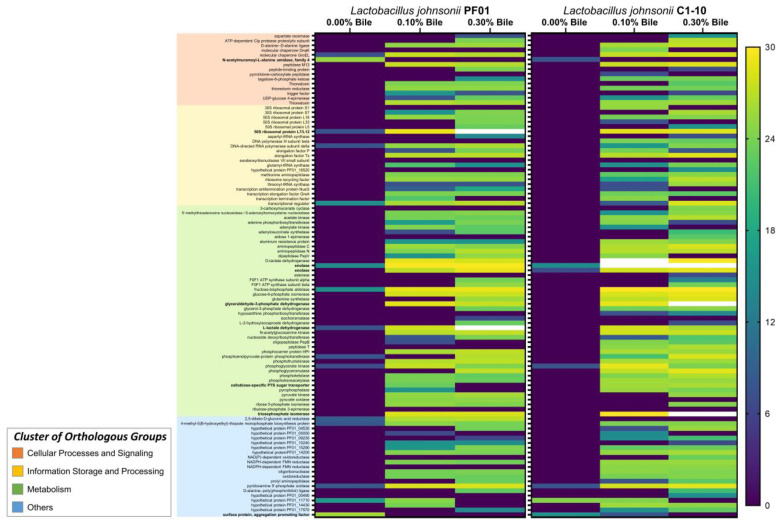
Expression heatmap analysis of proteins detected in both *Lactobacillus johnsonii* PF01 and C1-10. Proteins whose expression can be attributed as bile response are listed in Table 2. Corresponding *p* values (Tukey’s multiple comparisons) are in Supplementary Table S2.

Figure 3 shows a heatmap by strain and according to COG functional category. Five proteins were markedly upregulated by bile stress. In PF01, 50S ribosomal protein L7/L12 (RplL) and L-lactate dehydrogenase (Mdh) were highly upregulated. In C1-10, enolase expression was higher during bile stress than in the control (0.00%). In addition, in C1-10 glyceraldehyde-3-phosphate dehydrogenase (GapA) and triosephosphate isomerase (TpiA) were induced by bile.

**Figure 4:**
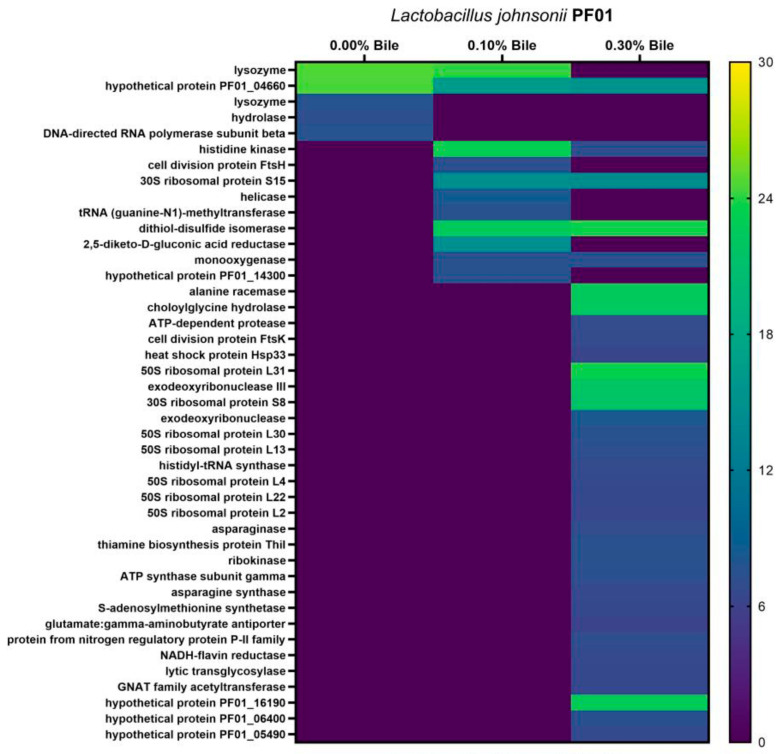

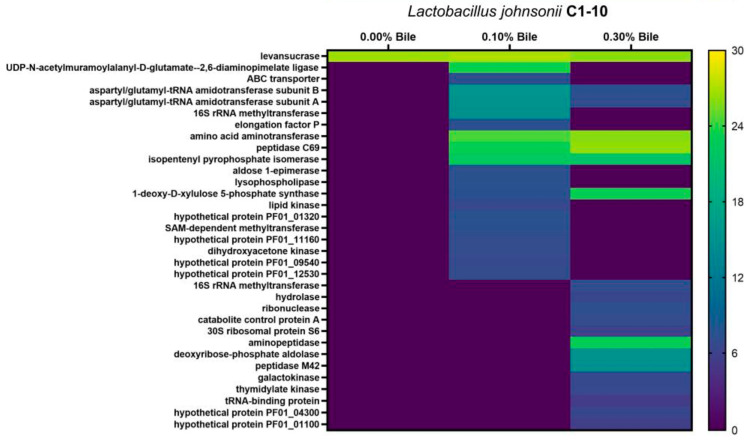
Expression heatmap analysis of proteins detected in either *Lactobacillus johnsonii* PF01 or C1-10. Corresponding *p* values (Tukey’s multiple comparisons) are in Supplementary Tables S3 and S4.

A heatmap of PF01 and C1-10 strain-specific proteins is shown in Figure 4. All of the proteins contributed to the survival of PF01 and C1-10 under bile stress. Therefore, we identified both bile-response proteins in *L*. *johnsonii* and analyzed the strain specificity of its exoproteome.

## 4. Discussion

### 4.1. Effect of Bile Stress on L. johnsonii Exoproteome

We have reported the extracellular proteins of *L*. *johnsonii* PF01 and C1-10 during mid-logarithmic growth [14]. In this study, we investigated their extracellular proteomes under bile stress. Cultures were grown until they reached the mid-logarithmic growth phase to exclude responses to other stress factors such as nutritional limitations and low pH during stationary phase [32,33,34].

Stress caused by bile induced extracellular secretion of proteins by PF01 and C1-10 (Figure 1). Because it survives in 0.30% to 0.50% bile [11], there is little possibility of cell lysis contributing to the protein count of PF01. Given that PF01 has bile salt hydrolases, these proteins expressed extracellularly give us another perspective to the PF01 bile response. PF01 does not undergo lysis, but rather creates a matrix of proteins outside the cell—proteins it did not secrete extracellularly in the absence of bile—which may have individual or collective functions that promote survival. The large number of common proteins between PF01 and C1-10 indicates that this phenomenon is common to the species.

### 4.2. Metabolic Adaptation to Support Protein Synthesis

Among the significant proteins common to PF01 and C1-10 in Figure 3, a large portion is metabolism related. Most are involved in glycolysis, such as fructose-bisphosphate aldolase, triosephosphate isomerase, phosphoglycerate kinase, glucose-6-phosphate isomerase, phosphoglyceromutase, and phosphofructokinase. Almost half (42%) are for carbohydrate transport and metabolism, and the others transport and metabolize amino acids, nucleotides, lipids, and inorganic ions. Among the common proteins showing strong white bands, four were involved in metabolism—enolase, L-lactate dehydrogenase (Mdh), glyceraldehyde-3-phosphate dehydrogenase (GapA), and triosephosphate isomerase (TpiA). Although metabolic proteins are localized intracellularly, they are reportedly present in the extracellular proteome of *L*. *johnsonii*. Like other members of the *Lactobacillus acidophilus* complex [35], *L*. *johnsonii* uses metabolic proteins to acquire nutrients they are incapable of synthesizing from the environment. Siciliano and Mazzeo [36] showed that the increased presence of metabolic proteins during bile stress suggests enhancement of extracellular nutrient breakdown. The resulting increased energy then sustains ATP-dependent processes in *L*. *johnsonii* in response to bile, e.g., optimization of protein synthesis. This is reflected by the parallel increases in the levels of proteins for translation, transcription, and ribosomal synthesis (information storage and processing) during bile exposure (Figure 2). Additionally, translation proteins were detected in the C1-10 exoproteome only after bile stress. This is supported by the significant upregulation of 50S ribosomal protein L7/L12 (RplL) for protein biosynthesis.

### 4.3. Cell-Wall Modifications as a Dose-Dependent Bile Response

Based on the heatmap analysis (Figure 3), among proteins common to the PF01 and C1-10 exoproteome under normal conditions, the expression of two proteins was abolished by bile—surface protein/aggregation promoting factor (*SPapf*) and N-acetylmuramoyl-L-alanine amidase (FlgJ). Both are N-terminally anchored membrane proteins predicted to be secreted by the general secretion pathway (Sec translocase/signal peptidase I) based on a bioinformatics analysis (LocateP v2.0 and SignalP4.0). In addition, their levels in the PF01 exoproteome were highest under normal conditions. Therefore, their absence under bile stress suggests that *L*. *johnsonii* manipulates its membrane structure/components as a response.

Other proteins with similar predicted secretion pathways (Table 3) and expression levels (Figure 3 and Figure 4) were similar to FlgJ. PF01 lysozyme (Acm) and hydrolase (Spr), which are involved in cell wall/membrane/envelope biogenesis, were secreted extracellularly via the Sec-(SPI) pathway under normal conditions (Figure 4). Neither protein was detected during bile treatment. The proteins secreted by PF01 changed as a function of bile concentration. Bile at 0.10% induced the secretion of cellobiose-specific PTS sugar transporter (CelA; carbohydrate transport and metabolism) and the cell division protein FtsH (post-translational modification). However, bile at 0.30% abolished the secretion of these two proteins and induced that of four other proteins—asparaginase (AnsB) and glutamate:gamma-aminobutyrate antiporter (PotE) (amino acid transport and metabolism), the cell division protein FtsK (cell cycle control), and lytic transglycosylase (LtgG) (peptidoglycan remodeling) [37]. These results suggest that PF01 undergoes cell wall modifications during its response to bile stress.

In the C1-10 exoproteome, only FlgJ secretion was abolished by bile. *SPapf* and C1-10 hypothetical protein (C1-10_104) secretion was abolished by 0.30% bile. As in PF01, secreted proteins were induced by 0.10% bile (cellobiose-specific PTS sugar transporter CelA [carbohydrate transport and metabolism]) and a hypothetical protein (C1-10_30). However, CelA secretion was not abolished by 0.30% bile. Therefore, C1-10 exhibits delayed the onset of the next cell wall modification, possibly due to its different bile response mechanism. Furthermore, the significant secretion of CelA by both strains suggests a role in the bile stress response. Interestingly, upregulation of CelA during bile stress was detected in the global proteomes of *L*. *johnsonii* PF01 and *L*. *paracasei* L9 [11,38].

### 4.4. Cytoplasmic Proteins Function as a Bile-Stress Protective Matrix

Although most proteins are predicted to be cytoplasmic/intracellular (Table 1), because the samples were supernatants, the data cannot indicate the intracellular metabolic pathway induced. For this reason, we discuss only the extracellular functions of some proteins, and hypothesize the functions of other proteins, during bile stress.

Numerous cytoplasmic proteins can be detected extracellularly under certain circumstances, e.g., during stress [10,39]. It is possible that some classified as cytoplasmic proteins harbor domains—known or not—that prompt their secretion during bile stress, and thus, they may have extracellular functions. For example, enolase, a cytoplasmic protein, has been detected in studies of stress responses and is a cell-surface protein in other organisms [40]. In addition, it moonlights as a binding and adhesion protein, together with GapA [41]. Similar moonlighting function can be detected for enolase, GapA, Mdh, TpiA, and RplL of *L*. *johnsonii*. In addition, bile-induced strain-specific proteins may protect the cell (Figure 4). Such proteins in PF01 include histidine kinase (COG1596), 30S ribosomal protein S15 (RpsO), and dithiol-disulfide isomerase (FrnE); in C1-10, this includes aspartyl/glutamyl-tRNA amidotransferase subunit A and B (GatA and GatB), amino acid aminotransferase (COG0436), peptidase C69 (PepD), isopentyl pyrophosphate isomerase (LldD), and 1-deoxy-D-xylulose 5-phosphate synthase (Dxs). Notably, the C1-10-specific proteins also have metabolic functions.

## 5. Conclusions

Extracellular proteins mediate survival responses to stress factors in the host gastrointestinal tract. The expression, regulation, and extracellular secretion of proteins mediate the response to bile stress. In *L*. *johnsonii*, bile stress-induced secretion of metabolism-related proteins as well as those for information storage and processing (translation, transcription, and ribosome synthesis) indicates that the bacterium adjusts its physiology in response to stress. Although most of these proteins are cytoplasmic and have no extracellular function, the marked changes in *L*. *johnsonii* secreted proteins suggests that they have functions in cell-wall modification as well as moonlighting functions in stress responses. The discovered extracellular proteins will be studied further for specific pathway analyses in line with the investigation of PF01 and C1-10 as potential probiotics.

## Figures and Tables

**Table 1:** Classification of all proteins from *Lactobacillus johnsonii* exoproteomes based on LocateP v2.0.

Classification	*Lactobacillus johnsonii*	
	PF01	C1-10
**Cellular Destination**		
Cytoplasmic	116	123
Membrane	8	4
Cell Wall	2	2
Extracellular	3	0
**Subcellular Localization**		
Intracellular	116	123
N-terminally anchored	4	4
Lipid-anchored	0	0
LPXTG Cell-wall anchored	2	2
Multi-transmembrane	4	0
Secretory	3	0
**Localization Class**		
Cytoplasm	114	117
Inner membrane	3	0
Periplasm	1	1
Secreted	11	11

**Table 2:** Putative proteins with bile response roles in both *Lactobacillus johnsonii* PF01 and C1-10 exoproteome based on significant difference in expression.

Effect of Bile on Expression (Treatment)	Locus Tag (PF01 Genome)	Protein Name	COG Gene	Detected in Other Bacteria during Bile Stress	Reference(s)
Upregulation (0.00%, 0.10%, and 0.30% bile)	PF01_08830	enolase	Eno	*Lactobacillus rhamnosus* GG; *Bifidobacterium longum, Enterococcus faecalis* V583, *Lactobacillus kefiranofaciens* M1	[8,15,21,22]
	PF01_13820	phosphoglycerate kinase	Pgk	*L. rhamnosus* GG, *B. longum* BBMN68, *B. longum* NCIMB 8809	[15,23,24]
	PF01_07580	pyridoxamine 5′-phosphate oxidase	COG3576		
Induction and upregulation (0.10% and 0.30% bile)	PF01_04460	50S ribosomal protein L7/L12	RplL		
	PF01_12550	5′-methylthioadenosine/S-adenosylhomocysteine nucleosidase	Pfs		
	PF01_13430	acetate kinase	ackA	*B. longum* BBMN68; *B. longum* NCIMB 8809	[23,24]
	PF01_08750	adenine phosphoribosyltransferase	Apt		
	PF01_18670	aluminum resistance protein	COG4100		
	PF01_04210	aminopeptidase C	PepC	*Bifidobacterium breve* UCC2003	[25]
	PF01_07460	aminopeptidase N	PepN		
	PF01_01440	D-alanine–D-alanine ligase	DdlA		
	PF01_00770	D-lactate dehydrogenase	LdhA	*L. kefiranofaciens* M1	[22]
	PF01_02700	DNA-directed RNA polymerase subunit delta	RpoE		
	PF01_03440	elongation factor P	FusA	*Campylobacter jejunii; B. longum* NCIMB 8809	[24,26]
	PF01_08410	elongation factor Ts	Tsf	*B. longum* BBMN68, *L. kefiranofaciens* M1	[22,23]
	PF01_13800	enolase	Eno	*L. rhamnosus* GG; *B. longum, E. faecalis* V583, *L. kefiranofaciens* M1	[8,15,21,22]
	PF01_05530	fructose-bisphosphate aldolase	Fba		
	PF01_13280	glucose-6-phosphate isomerase	Pgi		
	PF01_04250	glutamyl-tRNA synthase	GlnS		
	PF01_13830	glyceraldehyde-3-phosphate dehydrogenase	GapA	*B. longum* BBMN68, *B. longum* NCIMB 8809	[23,24,27]
	PF01_15290	hypothetical protein PF01_15290/C1-10_153	COG3679		
	PF01_03250	L-lactate dehydrogenase	Mdh	*L. rhamnosus* GG, *L. kefiranofaciens* M1	[15,22]
	PF01_07330	methionine aminopeptidase	Map	*B. longum* NCIMB 8809	[24]
	PF01_04920	molecular chaperone GroEL	GroL	*L. rhamnosus* GG, *Bacillus cereus* ATCC 14570; *B. longum, Lactobacillus casei* Zhang, *L. kefiranofaciens* M1, *Listeria monocytogenes*	[8,15,22,28,29,30]
	PF01_16360	N-acetylglucosamine kinase	COG2971		
	PF01_17540	NADPH-dependent FMN reductase	COG0431		
	PF01_01610	nucleoside deoxyribosyltransferase	COG3613		
	PF01_05020	oligoribonuclease	COG0618	*B. longum* BBMN68	[23]
	PF01_16660	oxidoreductase	COG2461		
	PF01_01930	peptidase M13	PepO		
	PF01_14360	phosphocarrier protein HPr	FruB	*L. rhamnosus* GG, *E. faecalis* V583	[15,21]
	PF01_17320	phosphofructokinase	FruK	*L. casei* Zhang, *L. monocytogenes*	[29,30]
	PF01_06630	phosphoketolase	COG3957		
	PF01_13700	phosphotransacetylase	Pta		
	PF01_08430	ribosome recycling factor	Frr	*B. longum* NCIMB 8809	[24]
	PF01_15140	thioredoxin	TrxA	*B. cereus* ATCC 14570, *E. faecalis* V583	[21,28]
	PF01_14000	thioredoxin reductase	TrxB	*B. cereus* ATCC 14570	[28]
	PF01_14910	threonyl-tRNA synthase	ThrS		
	PF01_14660	transcription elongation factor GreA	GreA	*B. longum* BBMN68	[23]
	PF01_11380	transcriptional regulator	HimA	*L. rhamnosus* GG; *E. faecalis* V583; *B. breve* UCC2003	[15,21,25]
	PF01_13810	triosephosphate isomerase	TpiA	*L. kefiranofaciens* M1	[22]
	PF01_16120	UDP-glucose 4-epimerase	GalE	*B. longum NCIMB* 8809	[24]
Stop expression (0.00% only)	PF01_02040	N-acetylmuramoyl-L-alanine amidase, family 4	FlgJ	*L. monocytogenes*	[30]
	PF01_15900	surface protein, aggregation promoting factor		*Lactobacillus acidophilus* NCFM	[31]

**Table 3:** Proteins with predicted secretion pathways based on LocateP v2.0 and SignalP4.0.

Locus Tag	*Lactobacillus johnsonii*		COG Gene	Secretion Pathway
	PF01	C1-10		
PF01_15900	surface protein, aggregation promoting factor			Sec-(SPI), Possibly Tat
PF01_02040	N-acetylmuramoyl-L-alanine amidase, family 4		FlgJ	Sec-(SPI), Possibly Tat
PF01_02390	cellobiose-specific PTS sugar transporter		CelA	Sec-(SPI)
PF01_04660	hypothetical protein PF01_04660		Smc	Sec-(SPI)
PF01_11380	transcriptional regulator		HimA	Possibly Tat/Sec-(SPI)
PF01_02050	lysozyme		Acm	Sec-(SPI)
PF01_17030	hydrolase		Spr	Sec-(SPI)
PF01_03320	cell division protein FtsH		FtsH	Sec-(SPI)
PF01_08990	asparaginase		AnsB	Sec-(SPI)
PF01_14190	cell division protein FtsK		FtsK	Sec-(SPI)
PF01_07170	lytic transglycosylase		LtgG	Sec-(SPI)
PF01_00790	glutamate:gamma-aminobutyrate antiporter		PotE	Sec-(SPI)
PF01_13400		levansucrase	SacC	Possibly Tat/Sec-(SPI)
PF01_11710		hypothetical protein C1-10_104		Possibly Tat/Sec-(SPI)
PF01_01320		hypothetical protein C1-10_30	COG4086	Sec-(SPI)
PF01_08830		enolase	Eno	Possibly Tat/No Pathway
PF01_13800		enolase	Eno	Possibly Tat/No Pathway
PF01_07580		pyridoxamine 5′-phosphate oxidase	COG3576	Possibly Tat/No Pathway
PF01_13820		phosphoglycerate kinase	Pgk	Possibly Tat/No Pathway

## Data Availability

The data presented in this study are available in (Ljohnsonii bile exoproteome (raw) Supplementary Materials).

## Supplementary Materials

The following are available online at https://www.mdpi.com/2227-7382/9/1/10/s1, Table S1: All proteins common between the two *Lactobacillus johnsonii* exoproteomes, grouped according to the effects of bile on protein expression, Table S2: Corresponding adjusted *p* values based on Tukey’s multiple comparison test used for expression heatmap analysis of common *Lactobacillus johnsonii* proteins, Table S3: Corresponding adjusted *p* values values based on Tukey’s multiple comparison test used for expression heatmap analysis of *Lactobacillus johnsonii* PF01 proteins, Table S4: Corresponding adjusted *p* values values based on Tukey’s multiple comparison test used for expression heatmap analysis of *Lactobacillus johnsonii* C1-10 proteins. Figure S1: Significant positive correlation between bile concentration and protein expression in *Lactobacillus johnsonii* PF01 and C1-10 during bile treatment.

## Abbreviations

Acm	lysozyme
ANOVA	analysis of variance
AnsB	asparaginase
ATP	adenosine triphosphate
BSH	bile salt hydrolase
CelA	cellobiose-specific PTS sugar transporter
COG	Cluster of Orthologous Groups
FlgJ	N-acetylmuramoyl-L-alanine amidase
GapA	glyceraldehyde-3-phosphate dehydrogenase
GI	Gastrointestinal tract
GRAS	Generally Regarded As Safe
HESI	heated electrospray ionization
LFQ	label-free quantification
LtgG	lytic transglycosylase
Mdh	L-lactate dehydrogenase
MS	mass spectrometry
Pgk	phosphoglycerate kinase
PotE	glutamate:gamma-aminobutyrate antiporter
PTS	phosphotransferase system
Pyr	pyridoxamine 5′-phosphate oxidase
RplL	50S ribosomal protein L7/L12
SPapf	surface protein/aggregation promoting factor
Spr	hydrolase
TpiA	triosephosphate isomerase
UHPLC	ultra-high-performance liquid chromatography

## References

[1] FAO/WHO (2002). Guidelines for the Evaluation of Probiotics in Food.

[2] Lebeer S., Vanderleyden J., De Keersmaecker S.C.J. (2008). Genes and Molecules of Lactobacilli Supporting Probiotic Action. Microbiol. Mol. Biol. Rev..

[3] Reid G. (1999). The Scientific Basis for Probiotic Strains of Lactobacillus. Appl. Environ. Microbiol..

[4] Begley M., Gahan C.G.M., Hill C. (2005). The Interaction between Bacteria and Bile. FEMS Microbiol. Rev..

[5] Waar K., van der Mei H.C., Harmsen H.J.M., Degener J.E., Busscher H.J. (2002). Adhesion to Bile Drain Materials and Physicochemical Surface Properties of Enterococcus Faecalis Strains Grown in the Presence of Bile. Appl. Environ. Microbiol..

[6] Pumbwe L., Skilbeck C.A., Nakano V., Avila-Campos M.J., Piazza R.M.F., Wexler H.M. (2007). Bile Salts Enhance Bacterial Co-Aggregation, Bacterial-Intestinal Epithelial Cell Adhesion, Biofilm Formation and Antimicrobial Resistance of Bacteroides Fragilis. Microb. Pathog..

[7] Khaleghi M., Kermanshahi R.K., Yaghoobi M.M., Zarkesh-Esfahani S.H., Baghizadeh A. (2010). Assessment of Bile Salt Effects on S-Layer Production, Slp Gene Expression and Some Physicochemical Properties of Lactobacillus Acidophilus ATCC 4356. J. Microbiol. Biotechnol..

[8] Ruiz L., Margolles A., Sánchez B. (2013). Bile Resistance Mechanisms in Lactobacillus and Bifidobacterium. Front. Microbiol..

[9] Sánchez B., Urdaci M.C., Margolles A. (2010). Extracellular Proteins Secreted by Probiotic Bacteria as Mediators of Effects That Promote Mucosa-Bacteria Interactions. Microbiology.

[10] Sánchez B., Bressollier P., Urdaci M.C. (2008). Exported Proteins in Probiotic Bacteria: Adhesion to Intestinal Surfaces, Host Immunomodulation and Molecular Cross-Talking with the Host. FEMS Immunol. Med. Microbiol..

[11] Lee J.Y., Pajarillo E.A.B., Kim M.J., Chae J.P., Kang D.-K. (2013). Proteomic and Transcriptional Analysis of Lactobacillus Johnsonii PF01 during Bile Salt Exposure by ITRAQ Shotgun Proteomics and Quantitative RT-PCR. J. Proteome Res..

[12] Valeriano V.D., Bagon B.B., Balolong M.P., Kang D.-K. (2016). Carbohydrate-Binding Specificities of Potential Probiotic Lactobacillus Strains in Porcine Jejunal (IPEC-J2) Cells and Porcine Mucin. J. Microbiol..

[13] Chae J.P., Valeriano V.D., Kim G.-B., Kang D.-K. (2013). Molecular Cloning, Characterization and Comparison of Bile Salt Hydrolases from Lactobacillus Johnsonii PF01. J. Appl. Microbiol..

[14] Bagon B.B., Valeriano V.D.V., Oh J.K., Pajarillo E.A.B., Cho C.-S., Kang D.-K. (2018). Comparative Exoproteome Analyses of *Lactobacillus* Spp. Reveals Species- and Strain-Specific Proteins Involved in Their Extracellular Interaction and Probiotic Potential. LWT.

[15] Koskenniemi K., Laakso K., Koponen J., Kankainen M., Greco D., Auvinen P., Savijoki K., Nyman T.A., Surakka A. (2011). Proteomics and Transcriptomics Characterization of Bile Stress Response in Probiotic Lactobacillus Rhamnosus GG. Mol. Cell. Proteom..

[16] Wiśniewski J.R., Zougman A., Nagaraj N., Mann M. (2009). Universal Sample Preparation Method for Proteome Analysis. Nat. Methods.

[17] Cox J., Mann M. (2008). MaxQuant Enables High Peptide Identification Rates, Individualized p.p.b.-Range Mass Accuracies and Proteome-Wide Protein Quantification. Nat. Biotechnol..

[18] Zhou M., Boekhorst J., Francke C., Siezen R.J. (2008). LocateP: Genome-Scale Subcellular-Location Predictor for Bacterial Proteins. BMC Bioinform..

[19] Petersen T.N., Brunak S., von Heijne G., Nielsen H. (2011). SignalP 4.0: Discriminating Signal Peptides from Transmembrane Regions. Nat. Methods.

[20] McHugh M.L. (2011). Multiple Comparison Analysis Testing in ANOVA. Biochem. Med..

[21] Bøhle L.A., Mathiesen G. (2010). Identification of Proteins Related to the Stress Response in Enterococcus Faecalis V583 Caused by Bovine Bile. Proteome Sci..

[22] Chen M.-J., Tang H.-Y., Chiang M.-L. (2017). Effects of Heat, Cold, Acid and Bile Salt Adaptations on the Stress Tolerance and Protein Expression of Kefir-Isolated Probiotic Lactobacillus Kefiranofaciens M1. Food Microbiol..

[23] An H., Douillard F.P., Wang G., Zhai Z., Yang J., Song S., Cui J., Ren F., Luo Y., Zhang B. (2014). Integrated Transcriptomic and Proteomic Analysis of the Bile Stress Response in a Centenarian-Originated Probiotic *Bifidobacterium Longum* BBMN68. Mol. Cell. Proteom..

[24] Sánchez B., Champomier-Vergès M.-C., Anglade P., Baraige F., de Los Reyes-Gavilán C.G., Margolles A., Zagorec M. (2005). Proteomic Analysis of Global Changes in Protein Expression during Bile Salt Exposure of Bifidobacterium Longum NCIMB 8809. J. Bacteriol..

[25] Ruiz L., Zomer A., O’Connell-Motherway M., van Sinderen D., Margolles A. (2012). Discovering Novel Bile Protection Systems in Bifidobacterium Breve UCC2003 through Functional Genomics. Appl. Environ. Microbiol..

[26] Fox E.M., Raftery M., Goodchild A., Mendz G.L. (2007). *Campylobacter Jejuni* Response to Ox-Bile Stress. FEMS Immunol. Med. Microbiol..

[27] Ruiz L., Couté Y., Sánchez B., de los Reyes-Gavilán C.G., Sanchez J.-C., Margolles A. (2009). The Cell-Envelope Proteome of Bifidobacterium Longum in an in Vitro Bile Environment. Microbiology.

[28] Kristoffersen S.M., Ravnum S., Tourasse N.J., Økstad O.A., Kolstø A.-B., Davies W. (2007). Low Concentrations of Bile Salts Induce Stress Responses and Reduce Motility in Bacillus Cereus ATCC 14570. JB.

[29] Wu R., Sun Z., Wu J., Meng H., Zhang H. (2010). Effect of Bile Salts Stress on Protein Synthesis of Lactobacillus Casei Zhang Revealed by 2-Dimensional Gel Electrophoresis. J. Dairy Sci..

[30] Payne A., Schmidt T.B., Nanduri B., Pendarvis K., Pittman J.R., Thornton J.A., Grissett J., Donaldson J.R. (2013). Proteomic Analysis of the Response of Listeria Monocytogenes to Bile Salts under Anaerobic Conditions. J. Med. Microbiol..

[31] Goh Y.J., Klaenhammer T.R. (2010). Functional Roles of Aggregation-Promoting-Like Factor in Stress Tolerance and Adherence of Lactobacillus Acidophilus NCFM. Appl. Environ. Microbiol..

[32] De Angelis M., Gobbetti M. (2004). Environmental Stress Responses in Lactobacillus: A Review. Proteomics.

[33] Kelly P., Maguire P.B., Bennett M., Fitzgerald D.J., Edwards R.J., Thiede B., Treumann A., Collins J.K., O’Sullivan G.C., Shanahan F. (2005). Correlation of Probiotic Lactobacillus Salivarius Growth Phase with Its Cell Wall-Associated Proteome. FEMS Microbiol. Lett..

[34] Cohen D.P.A., Renes J., Bouwman F.G., Zoetendal E.G., Mariman E., de Vos W.M., Vaughan E.E. (2006). Proteomic Analysis of Log to Stationary Growth Phase Lactobacillus Plantarum Cells and a 2-DE Database. Proteomics.

[35] Kullen M.J., Sanozky-Dawes R.B., Crowell D.C., Klaenhammer T.R. (2000). Use of the DNA Sequence of Variable Regions of the 16S RRNA Gene for Rapid and Accurate Identification of Bacteria in the Lactobacillus Acidophilus Complex. J. Appl. Microbiol..

[36] Siciliano R.A., Mazzeo M.F. (2012). Molecular Mechanisms of Probiotic Action: A Proteomic Perspective. Curr. Opin. Microbiol..

[37] Jenkins C.H., Wallis R., Allcock N., Barnes K.B., Richards M.I., Auty J.M., Galyov E.E., Harding S.V., Mukamolova G.V. (2019). The Lytic Transglycosylase, LtgG, Controls Cell Morphology and Virulence in Burkholderia Pseudomallei. Sci. Rep..

[38] Ma X., Wang G., Zhai Z., Zhou P., Hao Y. (2018). Global Transcriptomic Analysis and Function Identification of Malolactic Enzyme Pathway of Lactobacillus Paracasei L9 in Response to Bile Stress. Front. Microbiol..

[39] Lee Y.-J., Wang C. (2020). Proteomic Analysis Reveals the Temperature-Dependent Presence of Extracytoplasmic Peptidases in the Biofilm Exoproteome of Listeria Monocytogenes EGD-e. J. Microbiol..

[40] Ji H., Wang J., Guo J., Li Y., Lian S., Guo W., Yang H., Kong F., Zhen L., Guo L. (2016). Progress in the Biological Function of Alpha-Enolase. Anim. Nutr..

[41] Kainulainen V., Korhonen T.K. (2014). Dancing to Another Tune-Adhesive Moonlighting Proteins in Bacteria. Biology.

