# Supplementary material for: Exoproteome Perspective on the Bile Stress Response of Lactobacillus johnsonii
Source: Proteomes. 2021 Feb 10;9(1):10. doi: 10.3390/proteomes9010010 (PMC7931105; doi:10.3390/proteomes9010010)
Supplement: Supplementary file 1 [file proteomes-09-00010-s001.zip › Supplementary/Supplementary Materials.pdf]

Supplementary Materials

The data presented in this study are available in [Ljohnsonii bile exoproteome (raw) SUPP FILE.xlsx].

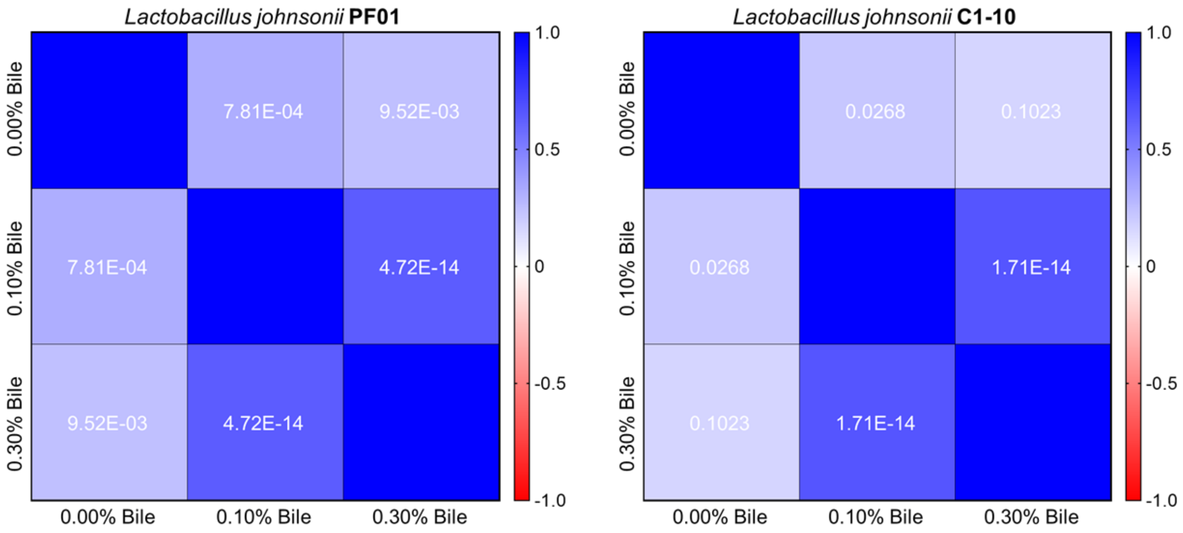

Supplementary Figure. Significant positive correlation between bile concentration and protein expression in *Lactobacillus johnsonii* PF01 and C1-10 during bile treatment; Correlation P values based on Pearson r are written in each cell.

Supplementary Table 1. All proteins common between the two *Lactobacillus johnsonii* exoproteomes, grouped according to the effects of bile on protein expression

**Effect of Bile Stress**	**Detected in Treatment**	**Locus Tag**	**Protein Name**
Bile stress stops expression	0.00% bile only	PF01_02040	N-acetylmuramoyl-L-alanine amidase, family 4
		PF01_15900	surface protein, aggregation promoting factor
Bile upregulates expression	0.00% to 0.30% bile	PF01_08830	enolase
		PF01_13820	phosphoglycerate kinase
		PF01_07580	pyridoxamine 5'-phosphate oxidase
Expressed in the presence of bile stress	0.10% to 0.30% bile	PF01_03860	4-methyl-5(B-hydroxyethyl)-thiazole monophosphate biosynthesis protein
		PF01_04460	50S ribosomal protein L7/L12
		PF01_12550	5'-methylthioadenosine nucleosidase / S-adenosylhomocysteine nucleosidase
		PF01_13430	acetate kinase
		PF01_08750	adenine phosphoribosyltransferase
		PF01_18670	aluminum resistance protein
		PF01_04210	aminopeptidase C
		PF01_07460	aminopeptidase N
		PF01_01440	D-alanine--D-alanine ligase
		PF01_00770	D-lactate dehydrogenase
		PF01_02700	DNA-directed RNA polymerase subunit delta
		PF01_03440	elongation factor P
		PF01_08410	elongation factor Ts
		PF01_13800	enolase
		PF01_05530	fructose-bisphosphate aldolase
		PF01_13280	glucose-6-phosphate isomerase
		PF01_04250	glutamyl-tRNA synthase
		PF01_13830	glyceraldehyde-3-phosphate dehydrogenase
		PF01_15290	hypothetical protein PF01_15290/C1-10_153
		PF01_03250	L-lactate dehydrogenase
		PF01_07330	methionine aminopeptidase
		PF01_04920	molecular chaperone GroEL
		PF01_16360	N-acetylglucosamine kinase
		PF01_17540	NADPH-dependent FMN reductase
		PF01_01610	nucleoside deoxyribosyltransferase
		PF01_05020	oligoribonuclease
		PF01_16660	oxidoreductase
		PF01_01930	peptidase M13
		PF01_14360	phosphocarrier protein HPr
		PF01_17320	phosphofructokinase
		PF01_06630	phosphoketolase
		PF01_13700	phosphotransacetylase
		PF01_08430	ribosome recycling factor
		PF01_15140	thioredoxin
		PF01_14000	thioredoxin reductase
		PF01_14910	threonyl-tRNA synthase
		PF01_14660	transcription elongation factor GreA
		PF01_11380	transcriptional regulator
		PF01_13810	triosephosphate isomerase
		PF01_16120	UDP-glucose 4-epimerase
Expressed in the presence of higher bile concentration	0.30% bile only	PF01_03430	30S ribosomal protein S7
		PF01_03620	50S ribosomal protein L18
		PF01_03580	50S ribosomal protein L5

		PF01_04710	adenylosuccinate synthetase
		PF01_17370	aspartate racemase
		PF01_09100	aspartyl-tRNA synthase
		PF01_13860	ATP-dependent Clp protease proteolytic subunit
		PF01_18100	D-alanine--poly(phosphoribitol) ligase
		PF01_13020	F0F1 ATP synthase subunit alpha
		PF01_13000	F0F1 ATP synthase subunit beta
		PF01_14560	glutamine synthetase
		PF01_14010	glycerol-3-phosphate dehydrogenase
		PF01_04530	hypothetical protein PF01_04530/C1-10_64
		PF01_09230	hypothetical protein PF01_09230/C1-10_126
		PF01_10240	hypothetical protein PF01_10240/C1-10_119
		PF01_17570	hypothetical protein PF01_17570/C1-10_173
		PF01_15880	L-2-hydroxyisocaproate dehydrogenase
		PF01_11370	peptide-binding protein
		PF01_14350	phosphoenolpyruvate-protein phosphotransferase
		PF01_02120	phosphoglyceromutase
		PF01_00840	prolyl aminopeptidase
		PF01_11500	pyruvate kinase
		PF01_06580	ribose 5-phosphate isomerase
		PF01_01530	tagatose-6-phosphate ketose
		PF01_05100	thioredoxin
		PF01_12290	trigger factor
Downregulated by higher bile concentration	0.00% to 0.10% bile	PF01_11710	hypothetical protein PF01_11710/C1-10_104
Bile-induced but higher bile concentration stops expression	0.10% bile only	PF01_03670	adenylate kinase
		PF01_05300	dipeptidase PepV
		PF01_05050	hypothetical protein PF01_05050/C1-10_70
		PF01_14430	hypothetical protein PF01_14430/C1-10_145
		PF01_17200	oligopeptidase PepB
		PF01_02390	cellobiose-specific PTS sugar transporter
		PF01_10310	pyrophosphatase
		PF01_04380	transcription antitermination protein NusG
		PF01_07780	transcription termination factor

Supplementary Table 2. Corresponding adjusted P values based on Tukey’s multiple comparison test used for expression heatmap analysis of common *Lactobacillus johnsonii* proteins

		Adjusted P-value					
		PF01			C1-10		
**Row**	**Protein ID**	**0.00% x 0.10%**	**0.00% x 0.30%**	**0.10% x 0.30%**	**0.00% x 0.10%**	**0.00% x 0.30%**	**0.10% x 0.30%**
1	aspartate racemase	>0.9999	0.146	0.146	>0.9999	0.0001	0.0001
2	ATP-dependent Clp protease proteolytic subunit	>0.9999	<0.0001	<0.0001	>0.9999	<0.0001	<0.0001
3	D-alanine--D-alanine ligase	<0.0001	<0.0001	0.9905	<0.0001	<0.0001	0.9388
4	molecular chaperone DnaK	>0.9999	>0.9999	>0.9999	<0.0001	>0.9999	<0.0001
5	molecular chaperone GroEL	<0.0001	<0.0001	0.9791	<0.0001	<0.0001	0.9614
6	N-acetylmuramoyl-L-alanine amidase, family 4	<0.0001	<0.0001	>0.9999	0.1021	0.1021	>0.9999
7	peptidase M13	<0.0001	<0.0001	0.9739	<0.0001	<0.0001	0.9596
8	peptide-binding protein	>0.9999	<0.0001	<0.0001	>0.9999	0.1796	0.1796
9	pyrrolidone-carboxylate peptidase	>0.9999	>0.9999	>0.9999	0.1617	>0.9999	0.1617
10	tagatose-6-phosphate ketose	>0.9999	0.0006	0.0006	<0.0001	<0.0001	0.9743
11	Thioredoxin	<0.0001	<0.0001	0.9997	>0.9999	<0.0001	<0.0001
12	thioredoxin reductase	<0.0001	<0.0001	0.9996	<0.0001	<0.0001	>0.9999
13	trigger factor	0.0003	0.1243	0.1245	>0.9999	0.1983	0.1983
14	UDP-glucose 4-epimerase	0.1202	<0.0001	0.0005	0.0001	<0.0001	0.099
15	thioredoxin	<0.0001	<0.0001	0.9865	<0.0001	<0.0001	0.9954
16	30S ribosomal protein S1	>0.9999	>0.9999	>0.9999	<0.0001	>0.9999	<0.0001
17	30S ribosomal protein S7	0.0003	<0.0001	0.0678	>0.9999	<0.0001	<0.0001
18	50S ribosomal protein L18	<0.0001	<0.0001	0.9509	<0.0001	<0.0001	0.9818
19	50S ribosomal protein L33	>0.9999	<0.0001	<0.0001	0.0988	>0.9999	0.0988
20	50S ribosomal protein L5	>0.9999	<0.0001	<0.0001	>0.9999	0.0002	0.0002
21	50S ribosomal protein L7/L12	<0.0001	<0.0001	0.9714	<0.0001	<0.0001	0.9999
22	aspartyl-tRNA synthase	>0.9999	0.0013	0.0013	>0.9999	0.1564	0.1564
23	DNA polymerase III subunit beta	>0.9999	>0.9999	>0.9999	<0.0001	<0.0001	0.9505
24	DNA-directed RNA polymerase subunit delta	<0.0001	<0.0001	0.9538	<0.0001	<0.0001	0.0668
25	elongation factor P	0.1478	<0.0001	0.0001	<0.0001	0.1097	<0.0001
26	elongation factor Ts	<0.0001	<0.0001	>0.9999	<0.0001	<0.0001	0.8893
27	exodeoxyribonuclease VII small subunit	>0.9999	>0.9999	>0.9999	>0.9999	>0.9999	>0.9999
28	glutamyl-tRNA synthase	<0.0001	0.0005	0.2364	0.0002	<0.0001	0.0525
29	hypothetical protein PF01_16520/C1-10_163	>0.9999	>0.9999	>0.9999	>0.9999	0.0003	0.0003
30	methionine aminopeptidase	<0.0001	<0.0001	>0.9999	<0.0001	<0.0001	0.8406
31	ribosome recycling factor	<0.0001	<0.0001	0.9982	<0.0001	<0.0001	0.0235
32	threonyl-tRNA synthase	0.1576	0.1365	0.9972	0.1395	<0.0001	<0.0001
33	transcription antitermination protein NusG	0.063	<0.0001	0.0685	0.124	>0.9999	0.124
34	transcription elongation factor GreA	<0.0001	<0.0001	0.9997	<0.0001	<0.0001	0.9668
35	transcription termination factor	<0.0001	>0.9999	<0.0001	<0.0001	>0.9999	<0.0001
36	transcriptional regulator	0.0174	0.0095	0.9779	0.0772	<0.0001	<0.0001
37	3-carboxymuconate cyclase	>0.9999	>0.9999	>0.9999	>0.9999	<0.0001	<0.0001
38	5'-methylthioadenosine nucleosidase / S-adenosylhomocysteine nucleosidase	<0.0001	<0.0001	0.9855	0.0001	<0.0001	0.0394
39	acetate kinase	<0.0001	<0.0001	0.9898	<0.0001	<0.0001	0.8693
40	adenine phosphoribosyltransferase	0.0001	<0.0001	0.1556	<0.0001	0.1744	<0.0001
41	adenylate kinase	<0.0001	<0.0001	0.8947	0.0002	>0.9999	0.0002
42	adenylosuccinate synthetase	0.1695	<0.0001	0.0005	>0.9999	<0.0001	<0.0001
43	aldose 1-epimerase	>0.9999	>0.9999	>0.9999	>0.9999	<0.0001	<0.0001
44	aluminum resistance protein	0.0002	0.0004	0.9762	<0.0001	<0.0001	0.9329
45	aminopeptidase C	<0.0001	<0.0001	0.9586	<0.0001	<0.0001	0.8815
46	aminopeptidase N	<0.0001	<0.0001	0.8157	<0.0001	<0.0001	0.9992
47	dipeptidase PepV	0.0004	<0.0001	0.0345	<0.0001	>0.9999	<0.0001
48	D-lactate dehydrogenase	<0.0001	<0.0001	0.9815	<0.0001	<0.0001	0.9961
49	enolase	0.0003	0.0004	>0.9999	<0.0001	<0.0001	0.9993
50	enolase	<0.0001	<0.0001	0.8797	<0.0001	<0.0001	0.9698
51	esterase	>0.9999	>0.9999	>0.9999	>0.9999	0.1771	0.1771
52	F0F1 ATP synthase subunit alpha	>0.9999	<0.0001	<0.0001	>0.9999	0.0014	0.0014
53	F0F1 ATP synthase subunit beta	>0.9999	<0.0001	<0.0001	>0.9999	<0.0001	<0.0001
54	fructose-bisphosphate aldolase	0.0018	0.0014	0.9978	<0.0001	<0.0001	>0.9999
55	glucose-6-phosphate isomerase	<0.0001	<0.0001	0.9985	<0.0001	<0.0001	0.9775
56	glutamine synthetase	>0.9999	<0.0001	<0.0001	<0.0001	<0.0001	0.9327
57	glyceraldehyde-3-phosphate dehydrogenase	<0.0001	<0.0001	0.946	<0.0001	<0.0001	0.9361
58	glycerol-3-phosphate dehydrogenase	>0.9999	<0.0001	<0.0001	<0.0001	<0.0001	0.9409
59	hypoxanthine phosphoribosyltransferase	>0.9999	>0.9999	>0.9999	0.1236	0.0006	0.1771

60	isochorismatase	>0.9999	0.2343	0.2343	>0.9999	>0.9999	>0.9999
61	L-2-hydroxyisocaproate dehydrogenase	>0.9999	<0.0001	<0.0001	>0.9999	0.1581	0.1581
62	L-lactate dehydrogenase	<0.0001	<0.0001	0.8433	<0.0001	<0.0001	0.8758
63	N-acetylglucosamine kinase	<0.0001	<0.0001	0.9952	<0.0001	<0.0001	0.9975
64	nucleoside deoxyribosyltransferase	0.1192	<0.0001	0.0002	0.0759	<0.0001	0.0009
65	oligopeptidase PepB	0.163	>0.9999	0.163	<0.0001	<0.0001	0.859
66	peptidase T	>0.9999	>0.9999	>0.9999	<0.0001	<0.0001	0.8931
67	phosphocarrier protein HPr	<0.0001	<0.0001	0.974	<0.0001	<0.0001	0.9681
68	phosphoenolpyruvate-protein phosphotransferase	0.1311	<0.0001	<0.0001	<0.0001	<0.0001	0.1491
69	phosphofructokinase	<0.0001	<0.0001	0.9913	<0.0001	<0.0001	0.9835
70	phosphoglycerate kinase	<0.0001	<0.0001	0.7027	<0.0001	<0.0001	0.3676
71	phosphoglyceromutase	>0.9999	<0.0001	<0.0001	<0.0001	<0.0001	0.9123
72	phosphoketolase	<0.0001	<0.0001	0.9658	<0.0001	<0.0001	0.802
73	phosphotransacetylase	<0.0001	<0.0001	0.9901	<0.0001	<0.0001	0.9873
74	cellobiose-specific PTS sugar transporter	<0.0001	>0.9999	<0.0001	<0.0001	<0.0001	0.9885
75	pyrophosphatase	0.0004	>0.9999	0.0004	<0.0001	<0.0001	0.9138
76	pyruvate kinase	<0.0001	<0.0001	0.9712	>0.9999	<0.0001	<0.0001
77	pyruvate oxidase	<0.0001	<0.0001	0.9973	>0.9999	>0.9999	>0.9999
78	ribose 5-phosphate isomerase	<0.0001	<0.0001	0.9807	>0.9999	<0.0001	<0.0001
79	ribulose-phosphate 3-epimerase	>0.9999	>0.9999	>0.9999	>0.9999	>0.9999	>0.9999
80	triosephosphate isomerase	<0.0001	<0.0001	0.9998	<0.0001	<0.0001	0.9836
81	2,5-diketo-D-gluconic acid reductase	<0.0001	<0.0001	0.9933	>0.9999	>0.9999	>0.9999
82	4-methyl-5(B-hydroxyethyl)-thiazole monophosphate biosynthesis protein	<0.0001	0.0002	0.9769	<0.0001	<0.0001	0.9951
83	hypothetical protein PF01_04530/C1-10_64	>0.9999	<0.0001	<0.0001	0.1389	<0.0001	<0.0001
84	hypothetical protein PF01_05050/C1-10_70	0.203	>0.9999	0.203	0.0005	>0.9999	0.0005
85	hypothetical protein PF01_09230/C1-10_126	>0.9999	0.2352	0.2352	0.0004	<0.0001	0.2001
86	hypothetical protein PF01_10240/C1-10_119	0.0004	0.0009	0.984	>0.9999	0.1985	0.1985
87	hypothetical protein PF01_15290/C1-10_153	0.0003	<0.0001	0.0796	<0.0001	<0.0001	0.9941
88	hypothetical proteinPF01_14200/C1-10_82	<0.0001	<0.0001	0.9919	<0.0001	<0.0001	0.9802
89	NAD(P)-dependent oxidoreductase	>0.9999	>0.9999	>0.9999	>0.9999	<0.0001	<0.0001
90	NADPH-dependent FMN reductase	<0.0001	<0.0001	0.9864	<0.0001	<0.0001	0.9969
91	NADPH-dependent FMN reductase	>0.9999	>0.9999	>0.9999	<0.0001	<0.0001	0.9998
92	oligoribonuclease	<0.0001	<0.0001	0.9941	<0.0001	<0.0001	0.9877
93	oxidoreductase	<0.0001	<0.0001	0.9888	<0.0001	<0.0001	0.984
94	prolyl aminopeptidase	>0.9999	<0.0001	<0.0001	0.1183	<0.0001	0.0002
95	pyridoxamine 5'-phosphate oxidase	<0.0001	<0.0001	>0.9999	<0.0001	<0.0001	0.9868
96	D-alanine--poly(phosphoribitol) ligase	>0.9999	<0.0001	<0.0001	>0.9999	<0.0001	<0.0001
97	hypothetical protein PF01_00490/C1-10_24	>0.9999	>0.9999	>0.9999	>0.9999	0.0003	0.0003
98	hypothetical protein PF01_11710/C1-10_104	0.0969	0.0001	<0.0001	0.9982	<0.0001	<0.0001
99	hypothetical protein PF01_14430/C1-10_145	<0.0001	>0.9999	<0.0001	<0.0001	<0.0001	0.9723
100	hypothetical protein PF01_17570/C1-10_173	>0.9999	0.001	0.001	0.0002	<0.0001	0.1008
101	surface protein, aggregation promoting factor	<0.0001	<0.0001	>0.9999	0.1167	0.0001	0.087

Supplementary Table 3. Corresponding adjusted P values values based on Tukey's multiple comparison test used for expression heatmap analysis of *Lactobacillus johnsonii* PF01 proteins

		**Adjusted P-value**		
**Row**	**Protein ID**	**0.00% x 0.10%**	**0.00% x 0.30%**	**0.10% x 0.30%**
1	lysozyme	0.9956	<0.0001	<0.0001
2	hypothetical protein PF01_04660	0.2953	0.2251	0.9861
3	lysozyme	0.3422	0.3422	>0.9999
4	hydrolase	0.3927	0.3927	>0.9999
5	DNA-directed RNA polymerase subunit beta	0.3299	0.3299	>0.9999
6	histidine kinase	0.0001	0.4	0.0114
7	cell division protein FtsH	0.3843	>0.9999	0.3843
8	30S ribosomal protein S15	0.0232	0.0256	0.9993
9	helicase	0.2789	>0.9999	0.2789
10	tRNA (guanine-N1)-methyltransferase	0.36	>0.9999	0.36
11	dithiol-disulfide isomerase	0.0002	<0.0001	0.9851
12	2,5-diketo-D-gluconic acid reductase	0.0212	>0.9999	0.0212
13	monooxygenase	0.3527	0.3769	0.9989
14	hypothetical protein PF01_14300	0.3573	>0.9999	0.3573
15	alanine racemase	>0.9999	0.0002	0.0002
16	choloylglycine hydrolase	>0.9999	0.0002	0.0002
17	ATP-dependent protease	>0.9999	0.3992	0.3992
18	cell division protein FtsK	>0.9999	0.4395	0.4395
19	heat shock protein Hsp33	>0.9999	0.4892	0.4892
20	50S ribosomal protein L31	>0.9999	<0.0001	<0.0001
21	exodeoxyribonuclease III	>0.9999	0.0003	0.0003
22	30S ribosomal protein S8	>0.9999	0.0003	0.0003
23	exodeoxyribonuclease	>0.9999	0.3063	0.3063
24	50S ribosomal protein L30	>0.9999	0.3594	0.3594
25	50S ribosomal protein L13	>0.9999	0.394	0.394
26	histidyl-tRNA synthase	>0.9999	0.4224	0.4224
27	50S ribosomal protein L4	>0.9999	0.4432	0.4432
28	50S ribosomal protein L22	>0.9999	0.4656	0.4656
29	50S ribosomal protein L2	>0.9999	0.4747	0.4747
30	asparaginase	>0.9999	0.4083	0.4083
31	thiamine biosynthesis protein ThiI	>0.9999	0.3697	0.3697
32	ribokinase	>0.9999	0.3714	0.3714
33	ATP synthase subunit gamma	>0.9999	0.3935	0.3935
34	asparagine synthase	>0.9999	0.4468	0.4468
35	S-adenosylmethionine synthetase	>0.9999	0.4666	0.4666
36	glutamate:gamma-aminobutyrate antiporter	>0.9999	0.5108	0.5108
37	protein from nitrogen regulatory protein P-II family	>0.9999	0.4086	0.4086
38	NADH-flavin reductase	>0.9999	0.4438	0.4438
39	lytic transglycosylase	>0.9999	0.4625	0.4625
40	GNAT family acetyltransferase	>0.9999	0.4668	0.4668
41	hypothetical protein PF01_16190	>0.9999	0.0002	0.0002
42	hypothetical protein PF01_06400	>0.9999	0.3766	0.3766
43	hypothetical protein PF01_05490	>0.9999	0.4461	0.4461

Supplementary Table 4. Corresponding adjusted P values values based on Tukey's multiple comparison test used for expression heatmap analysis of *Lactobacillus johnsonii* C1-10 proteins

		Adjusted P-value		
**Row**	**Protein ID**	**0.00% x 0.10%**	**0.00% x 0.30%**	**0.10% x 0.30%**
1	levansucrase	0.9958	0.9954	0.9826
2	UDP-N-acetylmuramoylalanyl-D-glutamate--2,6-diaminopimelate ligase	<0.0001	>0.9999	<0.0001
3	ABC transporter	0.3773	>0.9999	0.3773
4	aspartyl/glutamyl-tRNA amidotransferase subunit B	0.0116	0.3629	0.277
5	aspartyl/glutamyl-tRNA amidotransferase subunit A	0.0152	0.3824	0.3033
6	16S rRNA methyltransferase	0.0165	>0.9999	0.0165
7	elongation factor P	0.3544	>0.9999	0.3544
8	amino acid aminotransferase	<0.0001	<0.0001	0.9625
9	peptidase C69	<0.0001	<0.0001	0.8348
10	isopentenyl pyrophosphate isomerase	0.0002	0.0002	0.9943
11	aldose 1-epimerase	0.349	>0.9999	0.349
12	lysophospholipase	0.3523	>0.9999	0.3523
13	1-deoxy-D-xylulose 5-phosphate synthase	0.3605	<0.0001	0.0108
14	lipid kinase	0.4326	>0.9999	0.4326
15	hypothetical protein C1-10_30	0.3466	>0.9999	0.3466
16	SAM-dependent methyltransferase	0.3649	>0.9999	0.3649
17	hypothetical protein C1-10_109	0.3969	>0.9999	0.3969
18	dihydroxyacetone kinase	0.3976	>0.9999	0.3976
19	hypothetical protein C1-10_123	0.428	>0.9999	0.428
20	hypothetical protein C1-10_99	0.4105	>0.9999	0.4105
21	16S rRNA methyltransferase	>0.9999	0.3802	0.3802
22	hydrolase	>0.9999	0.4527	0.4527
23	ribonuclease	>0.9999	0.3708	0.3708
24	catabolite control protein A	>0.9999	0.3891	0.3891
25	30S ribosomal protein S6	>0.9999	0.5066	0.5066
26	aminopeptidase	>0.9999	<0.0001	<0.0001
27	deoxyribose-phosphate aldolase	>0.9999	0.0141	0.0141
28	peptidase M42	>0.9999	0.0162	0.0162
29	galactokinase	>0.9999	0.421	0.421
30	thymidylate kinase	>0.9999	0.4259	0.4259
31	tRNA-binding protein	>0.9999	0.5769	0.5769
32	hypothetical protein C1-10_57	>0.9999	0.4739	0.4739
33	hypothetical protein C1-10_28	>0.9999	0.5317	0.5317
