# Supplementary figures and images for: Exoproteome Perspective on the Bile Stress Response of Lactobacillus johnsonii
Source: Proteomes. 2021 Feb 10;9(1):10. doi: 10.3390/proteomes9010010 (PMC7931105; doi:10.3390/proteomes9010010)

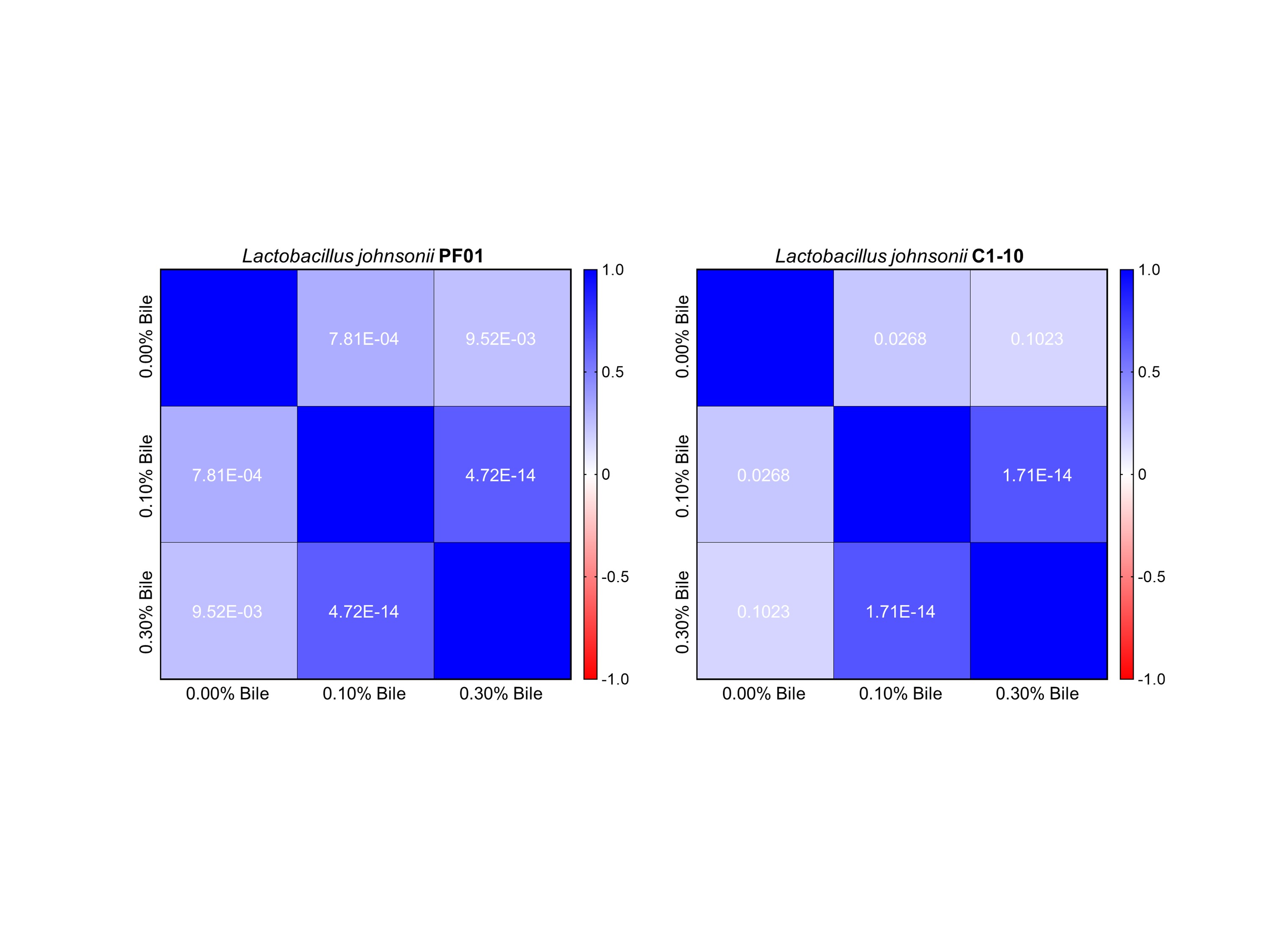

Supplement: Supplementary file 1 [file proteomes-09-00010-s001.zip › Supplementary/SuppFig.JPG]
